# Model-guided control of hippocampal discharges by local direct current stimulation

**DOI:** 10.1038/s41598-017-01867-1

**Published:** 2017-05-10

**Authors:** Faten Mina, Julien Modolo, Fanny Recher, Gabriel Dieuset, Arnaud Biraben, Pascal Benquet, Fabrice Wendling

**Affiliations:** 1 INSERM, Université de Rennes 1, LTSI, Rennes, F-35000 France; 2 Centre Hospitalier Universitaire Pontchaillou, Rennes, France

## Abstract

Neurostimulation is an emerging treatment for drug-resistant epilepsies when surgery is contraindicated. Recent clinical results demonstrate significant seizure frequency reduction in epileptic patients, however the mechanisms underlying this therapeutic effect are largely unknown. This study aimed at gaining insights into local direct current stimulation (LDCS) effects on hyperexcitable tissue, by i) analyzing the impact of electrical currents locally applied on epileptogenic brain regions, and ii) characterizing currents achieving an “anti-epileptic” effect (excitability reduction). First, a neural mass model of hippocampal circuits was extended to accurately reproduce the features of hippocampal paroxysmal discharges (HPD) observed in a mouse model of epilepsy. Second, model predictions regarding current intensity and stimulation polarity were confronted to *in vivo* mice recordings during LDCS (n = 8). The neural mass model was able to generate realistic hippocampal discharges. Simulation of LDCS in the model pointed at a significant decrease of simulated HPD (in duration and occurrence rate, not in amplitude) for cathodal stimulation, which was successfully verified experimentally in epileptic mice. Despite the simplicity of our stimulation protocol, these results contribute to a better understanding of clinical benefits observed in epileptic patients with implanted neurostimulators. Our results also provide further support for model-guided design of neuromodulation therapy.

## Introduction

Drug-resistant epilepsies are most often ‘partial’ or ‘focal’, i.e. characterized by an epileptogenic zone (EZ) that is relatively circumscribed in one of the two cerebral hemispheres. There is a large body of evidence indicating that the EZ is responsible for the generation of seizures, by altering the balance between excitatory and inhibitory processes in underlying neuronal networks^1^ that become “hyperexcitable”. This hyperexcitability, which is the hallmark of the EZ, is known to be at the origin of the many epileptiform events (such as interictal epileptic spikes^2, 3^, high-frequency oscillations^4–6^, electrographic seizures^7, 8^ typically observed in electrophysiological signals (scalp-EEG, depth-EEG, local field potentials).

Resective surgery is currently the only treatment capable of curing of some types of drug-resistant epilepsy^9^, provided that the EZ is (i) focal, (ii) clearly identified and (iii) can be safely removed^10^. However, surgical treatment can only be offered to 10–20% of drug-resistant patients^11^ as it may cause functional deficits. In addition, although often effective, epilepsy surgery still fails in a substantial percentage of patients despite extensive investigations^12^.

These considerations explain the high demand for therapeutic alternatives to resective surgery. A number of novel therapies have been pioneered in recent years^13^, like minimally invasive ablative procedures such as stereotactic laser ablation^14^ or stereotactic radiosurgery^15^. However, as in open resective surgery, contraindications still concern the majority of individuals due to a possibly low patient benefit vs. a significant risk of functional deficits.

In this context of refractory focal-onset epilepsy, neuromodulation techniques based on electrical stimulation have slowly developed over the past decades and they still represent a potentially-valuable therapeutic option^16^. Among available techniques, local invasive brain stimulation (i.e. using intracranial electrodes) has been applied to modulate the activity of epileptogenic networks^17^. Stimulation targets have included deep brain structures, such as the thalamic nuclei and the hippocampus^18^, as well as various cortical targets^19, 20^. Although it was shown to be effective in suppressing epileptic activity, local invasive stimulation is recognized to be largely empirical due to the absence of a rational definition of stimulation protocols, as indicated by inconsistent results among patients^21^. Recently, a responsive brain stimulation system has been developed^22, 23^ and tested in a large randomized controlled trial^24^. Although long-term results reported a seizure frequency decrease at 2 years (median percent reduction: 53%), this multicentric study concludes with two major points. First, it remains unknown how excitability is altered by electric stimulation. Second, there is still a large margin for improvement of therapeutic effects through the optimization of stimulation protocols.

The objective of this paper is to show that such an optimization can be significantly enhanced by a research approach combining computational models of epileptiform activity, biophysical models of electrical stimulation and experimental *in vivo* models of epilepsy. Specifically, we analyzed the polarization effects of electric fields induced by local DC stimulation in a neurophysiologically plausible computational model of hippocampal paroxysmal discharges (HPDs) classically observed in experimental models of epilepsy. These frequent sustained discharges (up to 60 seconds) have been hypothesized to be focal, non-convulsive seizures in epileptic mice^25^. The model was simulating activity from the dentate gyrus (DG) subfield of the hippocampus, suspected to be strongly involved in HPD generation and propagation throughout other hippocampus subfields^26^ (CA3, then CA1). The central role of the DG in HPD origin was confirmed in a recent study^27^ using optogenetics in the kainate model of epilepsy. From this computational model, we could predict the optimal stimulation polarity (anodal vs. cathodal, which is a critical issue in transcranial stimulation research^28, 29^) to reduce HPD rate and duration. Model predictions indicated that 1) HPD occurrence and duration decreased only in the cathodal stimulation condition (as opposed to anodal stimulation), and that 2) this effect is mainly mediated by the depolarization of slow, dendritic-targeting inhibitory interneurons. Then, we simulated the electric field generated by twisted wire electrodes at low intensities (on the order of 1 µA), and aimed at an implantation site slightly above the dentate gyrus to impact preferentially the numerous synaptic terminals of slow, dendritic-targeting interneurons located in this region. In order to verify these predictions, we performed *in vivo* recordings in n = 5 epileptic mice (kainate model), which confirmed a reversible, significant reduction of HPD duration and occurrence rate in the cathodal stimulation condition, as predicted by the model. The gained insights (differential effects on pyramidal cells and interneuron subtypes) as well as the limitations (monophasic stimulation) are discussed.

## Results

### *In silico* results

**Figure 1:**
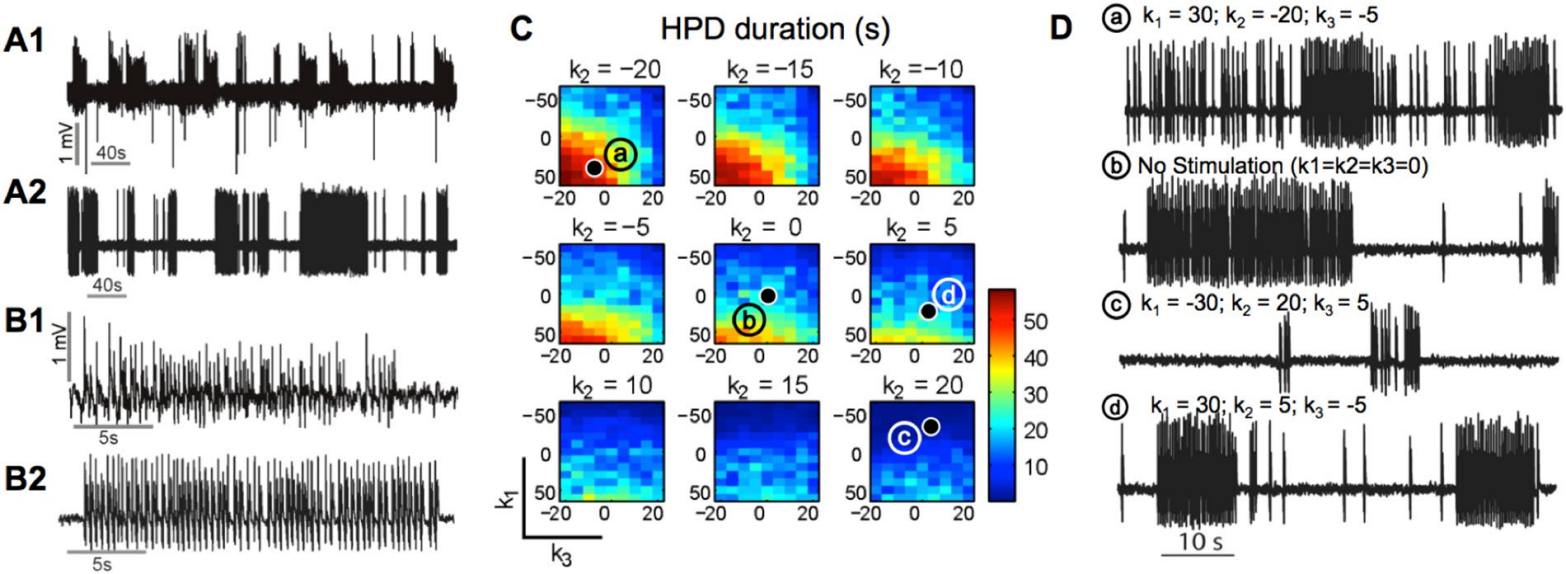
Effects of Local Direct Current Stimulation (LDCS) on HPD characteristics in the computational model. (**A1**) Example of experimentally recorded local field potential (LFP) recording in a KA mice, showing numerous HPD in the signal. (**A2**) Simulated LFP corresponding to (**A1**). (**B1**) Zoom on an experimentally recorded HPD. (**B2**) Zoom on the corresponding simulated HPD. (**C**) Mapping of different values for the stimulation coupling coefficients (k_1_, k_2_, k_3_) triplet, the color scale indicating the mean HPD duration for each triplet value. (**D**) Simulated LFP traces for the (a), (b), (c) and (d) triplet values.

The computational model was used in order to simulate DG dynamics, since experimental evidence points at a key role of the DG in HPD generation and propagation^3^. Realistic epileptic dynamics of the dentate gyrus (DG) was achieved in the model by tuning the parameters *A*, *B* and *G* – corresponding to EPSP/IPSP (Excitatory and Inhibitory Post-Synaptic Potentials, respectively) amplitudes of neuronal subpopulations – as well as the parameter *K* corresponding to the Input Noise Modulation Function (INMF) amplitude (see Materials and Methods section). Initial *A*, *B* and *G* values were set using previously published model activity maps^30^. Figure 1 presents a 400 s real intracerebral EEG (iEEG) signal segment (A1), representative of experimentally recorded HPD discharges in kainate mice and a simulated signal segment (A2) corresponding to the baseline condition in the absence of stimulation in the model. Experimentally recorded (B1) and simulated (B2) HPD occurring in these signal segments are also presented, illustrating that the model is capable of accurately capturing the dynamics of *in vivo* hippocampal epileptic activity.

As described in the Materials and Methods section, model output was quantified for each triplet of stimulation coupling coefficients (k_1_, k_2_, k_3_). The three parameters k_1_, k_2_ and k_3_ represent, in the model, the impact of the electric field on each neuronal type. In brief, they are used to describe the coupling between the electric field resulting from stimulation and the resulting membrane polarization at the neuronal level for each specific neuronal type. A linear model was assumed: at each neuronal subpopulation (e.g., granule cells, GC), the amount of membrane polarization is proportional to the stimulation current weighted by the corresponding coupling coefficient (e.g., k_1_ for GC cells, k_2_ for Interneuron Fast Somatic Inhibition, IFSI; and k_3_ for Interneuron Slow Dendritic Inhibition, ISDI). Note that the sign of these coupling coefficients accounts for the stimulation polarity (positive for anodal stimulation, negative for cathodal stimulation). We studied a fixed 3D parameter space limited by the intervals [k_1, min_ k_1, max_], [k_2, min_ k_2, max_] and [k_3, min_ k_3, max_]. Stimulation current intensity was fixed to 1 µA and limited to a duration of 50 s. Figure 1C depicts stimulation cartography in the predefined parameter space (k_1_, k_2_, k_3_). The value attributed to each triplet corresponds to the mean discharge duration of 5 stochastic simulations over one-minute windows. Effects corresponding to discharge intensity and number of detected peaks (not illustrated) followed the same pattern. This cartography shows that the highest levels of excitability in the model are correlated with positive high values of k_1_ (i.e., depolarization of GC) coupled to negative values of k_3_ (hyperpolarization of dendrite-projecting neurons) when k_2_ is significantly negative (≤−10, corresponding to hyperpolarization of soma-projecting neurons, lower left corners of individual maps). Similarly, when the value of k_3_ is significantly positive (≥10), slightly negative values of k_1_ seem sufficient to suppress epileptic activity. In conclusion, low-intensity DC stimulation effects are highly dependent on DC stimulation current impact on the impacted neuronal population types. More specifically, the maximal HPD duration decrease is obtained by the hyperpolarization of granule cells combined with the depolarization of dendritic-targeting interneurons. For illustration, Fig. 1D presents simulated signal segments (points (a), (b), (c) and (d) in the parameter space) corresponding to the baseline condition (panel (b)) and to two stimulations of opposite polarities in the parameter space corresponding to (a) and (c).

### *In vivo* results

#### Protocol 1 - Polarity-dependent effects

**Figure 2:**
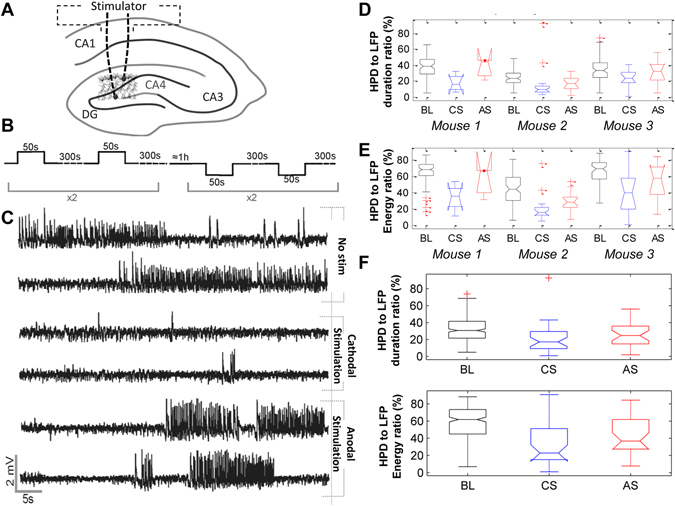
Differential effects of anodal and cathodal stimulation on HPD characteristics. (**A**) Illustration of the stimulation electrode tips position across the GC layer in the DG. (**B**) LDCS protocol followed to identify which stimulation polarity was efficient in decreasing tissue hyperexcitability. (**C**) Time series of recorded LFP during epochs of 1) no stimulation, 2) cathodal stimulation (CS), 3) anodal stimulation (AS). The significant reduction of HPD duration and occurrence during CS is noticeable. (**D**) Boxplot of the HPD to total recording duration ratio for the three tested animals, without stimulation, and during CS/AS. (**E**) same as (**D**) for the HPD to LFP energy ratio. (**F**) *Upper panel*: Pooled results on the three mice regarding the HPD to LFP duration ratio. *Lower panel*: same as (**F**) for the HPD to LFP energy ratio. Red crosses are data points outside the 1.5 * IQR (interquartile range) represented by the whiskers, as provided by Matlab (The Mathworks, USA). For each box, the central line is the median, while the lower/upper edges are the 25^th^ and 75^th^ data percentiles, respectively.

Local field potentials (LFP) signals recorded with/without LDCS were analyzed over a 60 s window. Stimulation responses were categorized given the two possible inverse stimulation polarities available using the GRASS Technologies S88X stimulator denoted AS (anodal stimulation) and CS (cathodal stimulation). The chosen stimulation target was the DG, based on its strategic position as the main input to the hippocampus, and on evidence that temporal seizures originate mostly from the enthorinal cortex, a major DG input^31^. The anode and the cathode were surgically placed from each side of the DG, with the anode deeper, with the objective to target, based on modeling predictions, dendritic-targeting GABAergic interneurons. Computed discharge characteristics were then displayed in boxplots for each mouse to illustrate their statistical significance. Figure 2C shows experimentally recorded LFP signals representative of effects observed in Protocol 1. The upper panel presents a typical baseline activity of interictal activity of a kainate mouse. The middle panel presents hippocampal activity in the same mouse, right after the end of a cathodal DC stimulation (CS), illustrating a considerable decrease in HPD frequency. Finally, the lower panel shows typical hippocampal activity after anodal stimulation cessation. No visible change in discharge characteristics was clearly correlated with this stimulation polarity.

Plotting the boxplots of normalized discharge characteristics (Fig. 2D and E) pointed at a quantifiable statistical significance in polarity-dependent stimulation effects. As shown in Fig. 2D, the percentage of HPD duration in the minute following the end of a cathodal stimulation was significantly decreased. Statistical significance was assessed using a single test, the Mann-Whitney non-parametric test (standard 5% significance level). This was especially noticeable for Mouse 1 and 2, in which discharge duration/unit time was divided at least by a factor two (mouse 1: from 22.5 ± 8,1 s before stimulation to 10.6 ± 6.7 s after stimulation, p = 0.011; mouse 2: from 14,4 ± 5.4 s before stimulation to 5.8 ± 2.5 s after stimulation, p < 0.001; mouse 3: from 19.6 ± 8.2 s before stimulation to 14.1 ± 6.4 s after stimulation, p = 0.023). However, no significantly visible effect was observed for anodal stimulation (mouse 1: 22.5 ± 8.4 after stimulation, p = 0.97; mouse 2: 10.1 ± 5.5 s after stimulation, p = 0.005; mouse 3: 19.2 ± 8.8 s after stimulation, p = 0.81). Similarly, Fig. 2F shows that the percentage intensity of detected HPDs (percentage of the signal’s total energy) was also decreased in the minute following cathodal stimulation cessation. Again, no significant effect was related to the opposite polarity (anodal stimulation). Boxplots showed that cathodal simulation systematically induced a significant decrease in discharge characteristics in the minute following stimulation; while anodal stimulation did not change discharge characteristics. We performed a Mann-Whitney statistical test in order to evaluate whether the effects of cathodal stimulation were significantly superior to those of anodal stimulation in term of HPD feature reduction. The test was used both at the individual level and at the group level (n = 3). In all animals, cathodal stimulation led to significant reduction in HPD duration as compared to anodal stimulation (p < 0.01 for each animal, p = 0.041 for the group). Similarly, a more pronounced reduction of the energy ratio was observed under CS (32.95 ± 21.49%), as compared to AS (42.74 ± 20.38%), with a baseline of 58.18 ± 18.2%. Statistical comparison (Mann-Whitney) of energy ratio values measured under both conditions indicated that this difference is significant (p = 0.029). As depicted in Fig. 2, boxplots showed that cathodal stimulation systematically induced a significant decrease in discharge characteristics in the minute following stimulation for every animal (see Fig. 2D and E) as well as for the group (Fig. 2F). Therefore, our results indicate a superior antiepileptic effect of cathodal stimulation, as quantified by considered HPD features (duration and energy ratio).

Figure 2F presents the corresponding simulated boxplot of cathodal and anodal stimulation in the model. In order to reproduce the experimental boxplot, we hypothesized that fast interneurons (IFSI; fast somatic-targeting interneurons) were always depolarized by the stimulation current. It has been actually reported that slow, dendritic-targeting interneurons (found in the hippocampal stratum lacunosum-moleculare layer) are easily depolarized under electrical stimulation independently from polarity^32^. Therefore, the depolarization of these interneurons could compensate granule cells depolarization during anodal stimulation, and reinforce the hyperpolarization effect during cathodal stimulation. In the model, under this hypothesis, we could reproduce the experimental dynamics observed under LDCS. The triplet of k_1_, k_2_, k_3_ values (30, 5, −5) was used for simulating cathodal stimulation while (−30, 5, 5) was used for simulating anodal simulation.

#### Protocol 2 - Time-dependent effects

**Figure 3:**
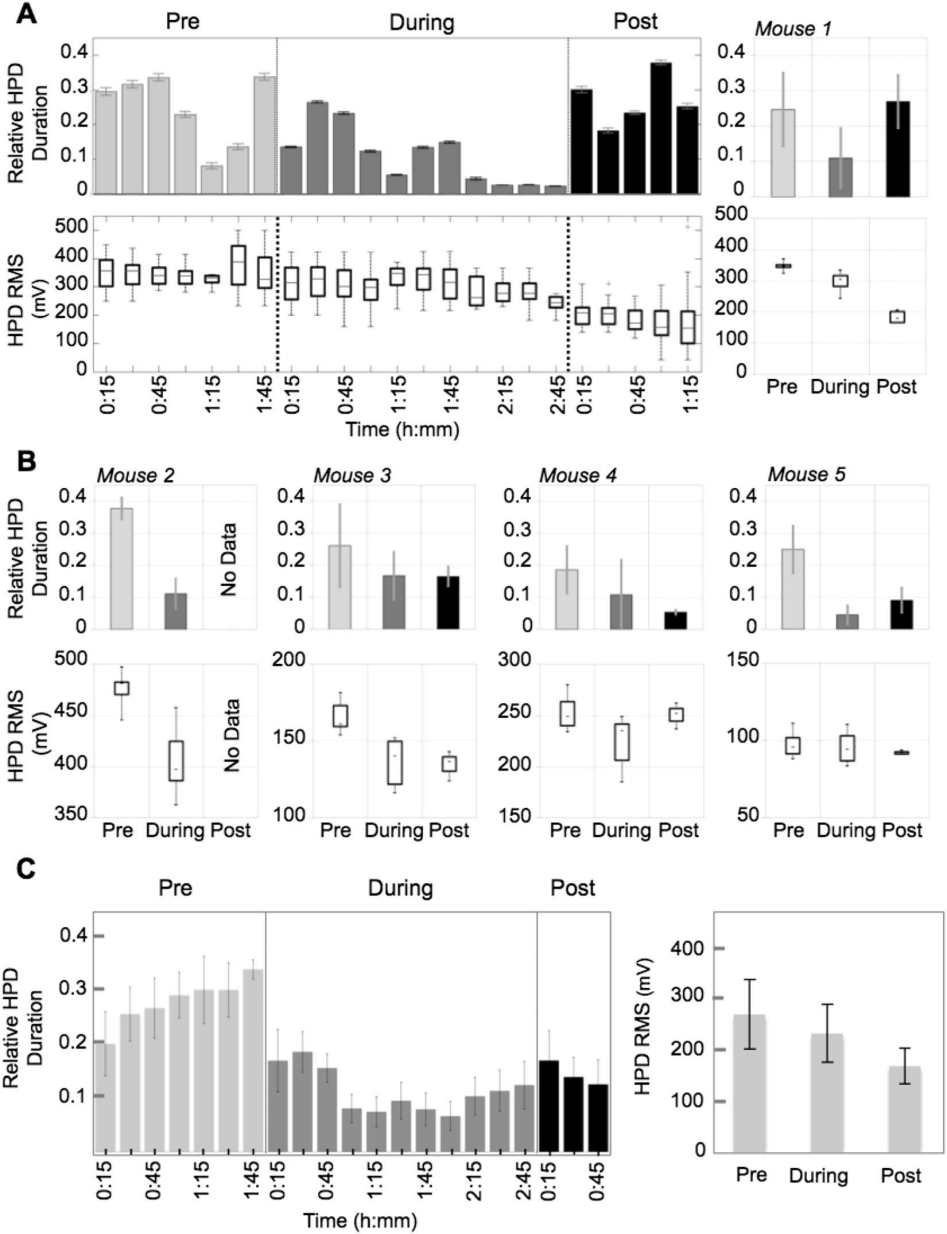
Effect of the cathodal stimulation on HPD occurrence and duration. (**A**) *Upper left*. Moving average of relative HPD duration over time, pre-, per- and post-stimulation. *Lower left*. Evolution of HPD RMS value over time, pre-, per- and post-stimulation. *Upper right*. Average relative HPD duration pre-, per- and post-stimulation for Mouse 1. *Lower right*. Average relative HPD duration pre-, per- and post-stimulation for Mouse 1. (**B**) *Upper row*. Average relative HPD duration pre-, per- and post-stimulation for Mouse 2, 3, 4 and 5. *Lower row*. Average relative HPD duration pre-, per- and post-stimulation for Mouse 2, 3, 4 and 5. (**C**) *Group data*: evolution, as a function of time, of the relative HPD duration averaged over the 5 animals (mouse 1–5). The relative HPD duration was defined as the HPD cumulated durations divided by the analysis window duration (chosen to be equal to 30 min.).

Since polarity-dependent results identified cathodal stimulation as the optimal protocol to decrease HPD occurrence and duration, we followed Protocol 1 to identify the time course of cathodal stimulation on HPD characteristics. Results from this protocol are presented in Fig. 3, where the relative HPD duration was defined, for a given interval of time, by the cumulated duration of HPD divided by the considered time of interval. This provided us with a normalized quantity indicating the proportion of time spent by the recorded brain area to generate HPD. Regarding HPD duration and amplitude, Fig. 3A illustrates a cumulative and reversible effect of LDCS stimulation epochs, since HPD duration gradually decreased over time and became negligible at the end of the stimulation session, before returning back to pre-stimulation levels. Figure 3B presents individual mice data regarding HPD duration and RMS value in each condition (pre-, during- and post-stimulation). RMS holds for “root-mean-square”, and is defined by the square root of the mean squared signal. Figure 3C (left panel) presents the time course of LDCS effects on HPD duration over the course of the experiment. On the right panel of Fig. 3C, a significant decrease (p < 0.001, Mann-Whitney test) of HPD RMS was observed during stimulation (230.9 ± 55.8 mV) as compared to baseline (267.7 ± 66.9 mV), and this decrease was confirmed further even post stimulation (168 ± 34.9 mV). Even though this observation is based on results obtained from a small number of animals (n = 5), it suggests that lasting effects are also induced in addition to acute stimulation effects, manifesting as a reduction in HPD amplitude even post-stimulation. Interestingly, this decrease in HPD amplitude is observed post-stimulation even if HPD duration has returned to pre-stimulation levels, suggesting that HPD duration and amplitude depend on distinct physiological processes/parameters.

Statistical analysis based on a single test (Mann-Whitney test) showed significant difference between the HPD duration measured during baseline condition and those measured during the stimulation sessions for each animal (mouse 1: from 0.24 ± 0.1 before stimulation to 0.1 ± 0.08 after stimulation, p = 0.012; mouse 2: from 0.37 ± 0.03 before stimulation to 0.11 ± 0.04 after stimulation, p < 0.001; mouse 3: from 0.25 ± 0.12 before stimulation to 0.16 ± 0.07 after stimulation, p = 0.022; mouse 4: from 0.18 ± 0.07 before stimulation to 0.1 ± 0.1 after stimulation, p = 0.032; mouse 5: from 0.24 ± 0.07 before stimulation to 0.04 ± 0.02 after stimulation, p = 0.0017), but also for the group (p < 0.001).

## Discussion

We studied LDCS effects on interictal epileptiform hippocampal discharges using a computational model of the hippocampus. The major originality of this work is that the computational model guided us towards an LDCS protocol (specific electrodes location based on defined neuroanatomical targets, stimulation polarity) able to suppress HPD *in silico*, which was then experimentally tested and validated *in vivo* through DG stimulation, motivated by the key role of this hippocampal subfield in epileptiform activity generation. The main finding of this study is that cathodal LDCS can be applied on the seizure-onset zone *in vivo*, in a region where granule cell dendrites have a significant preferred orientation and where synaptic terminals of dendritic-targeting GABAergic neurons are massively present (as suggested by model predictions), to induce a rapid decrease in epileptiform discharges both in the model and *in vivo*. Our stimulation electrodes were placed as follows: the first in the inner part of the GC layer, and the second at the most external part of the dentate gyrus to maximize stimulation impact on GC dendrites, in order to stimulate dendritic-targeting GABAergic neurons, based on model predictions. The modeling approach was based on an established model of hippocampal epileptiform discharges^33^, extended to simulate DG dynamics in two ways. The first computational extension was a phenomenological stochastic implementation of HPD occurrence where HPD duration and inter-HPD duration were considered as two independent random values. Another possible implementation could consist in using a Bayesian model of HPD occurrence where the aforementioned two variables are dependent, which can be inferred from reliability theory where it is considered that the system has a higher discharge probability when the time spent in the background state is longer. A similar approach using reliability theory was proposed for modeling iEMG signals^34^. However, our phenomenological implementation was sufficient for the scope of our study. In our model, the modified noise input is considered to originate from a DG subpopulation, projecting on the main DG population that generates HPD. The second computational extension was the inclusion in the original hippocampal model of electrical stimulation effects, which enabled studying LDCS effects on HPD duration and occurrence rate. Finally, our stimulation input model was based on the polarization of presynaptic neuronal elements by stimulation currents^18, 32, 35^, in line with recent results^36^ pointing at effects of transcranial DC stimulation on synaptic terminals. Interestingly, our model results point at the importance of depolarizing dendritic-projecting GABAergic interneurons with the DC stimulation to decrease HPD duration, which could actually compensate the decrease in dendritic GABAergic inhibition that has been reported in experimental models of epilepsy^37^. Interestingly, it has been recently shown that specific activation GABAergic interneurons of the DG by optogenetic was able to inhibit the propagation of seizures and largely rescue behavioural deficits in kainate-exposed animals^27^. Therefore, this recent study gives further support to our proposed mechanism based on the activation of dendrite-projecting GABAergic interneurons of the dentate gyrus due to LDCS.

A limitation of the present study is that we considered an *in vivo* DC stimulation protocol, which is not a viable technique for chronic neuromodulation. Nevertheless, this study brings evidence that pathological hyperexcitability can be modulated *in vivo* using a simple stimulation protocol. DC stimulation is well-known to induce electrolysis, since charge accumulation cannot be compensated by a pulse of opposite polarity preventing charge accumulation. In order to minimize tissue damage^38^ during long-term stimulation, a stimulation protocol should be charge-balanced (i.e. the integral of a stimulation pulse waveform must be equal to zero to avoid charge accumulation). Imbalanced stimulation protocols have been shown to induce neuronal damage quickly: Piallat *et al*.^39^ studied the volume of damaged tissue after monophasic stimulation and pointed that measurable lesions occur after only 5 minutes of monophasic stimulation; while biphasic stimulation delivered for several hours did not result in any damage. In order to address the issue of possible charge accumulation due to DC stimulation, which can ultimately result in tissue damage, future research efforts should focus on the translation of these results into clinically usable stimulation protocols, which have to be charge-balanced for safety reasons. Since our *in silico* results suggest that depolarizing the synaptic terminals of dendrite-projecting GABAergic interneurons is the mechanism underlying HPD reduction, this provides a starting point for the development of pulsed, charge-balanced stimulation protocols that could decrease pathological hyperexcitability with a well-defined neurophysiological target.

It should also be mentioned that the model used was not able to explicitly describe the interaction site between the induced electric field and neural elements. Indeed, the input to a given neuronal subpopulation consists in applying a sigmoid function to the total presynaptic potential. Somatic or synaptic terminals polarization cannot be distinguished in the model, since it is represented in the same way: if the soma is stimulated, it will slightly increase population firing rate through the sigmoid function; if synaptic terminals are stimulated, the neurotransmitter release rate will increase, also increasing the firing rate through the sigmoid function. Therefore, the model describes a presynaptic modulation of neuronal activity, without any *a priori* on the interaction site.

In this combined *in silico/in vivo* study, we have shown that local intracerebral cathodal DC stimulation can modulate epileptiform activity in a mouse model of epilepsy, guided by a biologically grounded model of hippocampal activity. While the effect of cathodal stimulation appears significantly superior to anodal stimulation, one limit is that our stimulation protocol investigating both polarities was not counterbalanced, therefore, an order effect cannot be excluded. Furthermore, due to the lasting effects of cathodal stimulation on HPD occurrence that we observed experimentally, there is a possibility that anodal stimulation actually increases HPD occurrence. Such possibility remains to be confirmed experimentally. In addition to this polarity-dependent effect, we have shown a cumulative, time-dependent effect leading to a significant reduction in HPD duration. Our data also suggests a possible lasting effect of the stimulation on HPD amplitude after stimulation cessation, which was however not predicted by the model in which no such potential lasting effects were implemented. HPD duration reduction was reversible, and required several stimulation blocks to take place. Taken together, these results suggest that a combination of acute and lasting effects underlie this modulation of epileptiform activity, consistently with the literature reporting neuromodulation effects in epilepsy. In our physiologically grounded model, this decrease in HPD duration during cathodal stimulation is explained by hyperpolarization of DG granule cells, along with a depolarization of slow, dendrite-projecting interneurons. This polarization was instantaneous in the model, whereas experimentally stimulation effects appear after some time, suggesting that acute depolarizing effects trigger a slower physiological process, possibly related to synaptic plasticity, ultimately resulting in HPD rate and duration decrease. Let us also mention that no plasticity-related mechanism was explicitly implemented to account for lasting effects. Furthermore, EEG data was simulated both during stimulation epochs and during epochs free of stimulation. In contrast, in our experimental setting, EEG data was not accessible during stimulation epochs themselves, due to the saturation of EEG amplifiers. Therefore, EEG data was only available, for the *in vivo* experiment, between stimulation sessions. One consequence is that acute effects of LDCS could only be assessed through the immediate epochs following the stimulation offset. Nevertheless, both simulated and experimental EEG data are still comparable since the experimentally observed post-stimulation HPD reduction is likely already present just before stimulation cessation, during the stimulation epoch that is accessible in the model. These observed lasting effects are likely due, at least in part, to other mechanisms not yet implemented in the model, such as changes in synaptic plasticity. One experimental observation in favour of this hypothesis is the reversible reduction in HPD occurrence rate. Indeed, this rate returns to baseline (pre-stimulation) value after the termination of stimulation, suggesting short-term plasticity changes that build up during stimulation and fade after stimulation. This interesting possibility, that requires extending the model to account for long-term changes in synaptic weights, will be the focus of future investigations.

Despite a small sample size, this combined experimental and theoretical study provides further mechanistic insights on local electrical stimulation of epileptogenic networks, which will be explored in future works aiming to use charge-balanced neurostimulation protocols achieving similar reductions in brain tissue excitability. Furthermore, our results support that model-guided neuromodulation protocols have the potential to facilitate the transfer to clinically usable protocols by suggesting optimal stimulation sites and targets based on specific neuroanatomical and neurophysiological characteristics. Following such approaches might help to move from empiric neuromodulation protocols to rationally designed neuromodulation therapies.

## Materials and Methods

### *In vivo* data and HPD statistics

**Figure 4:**
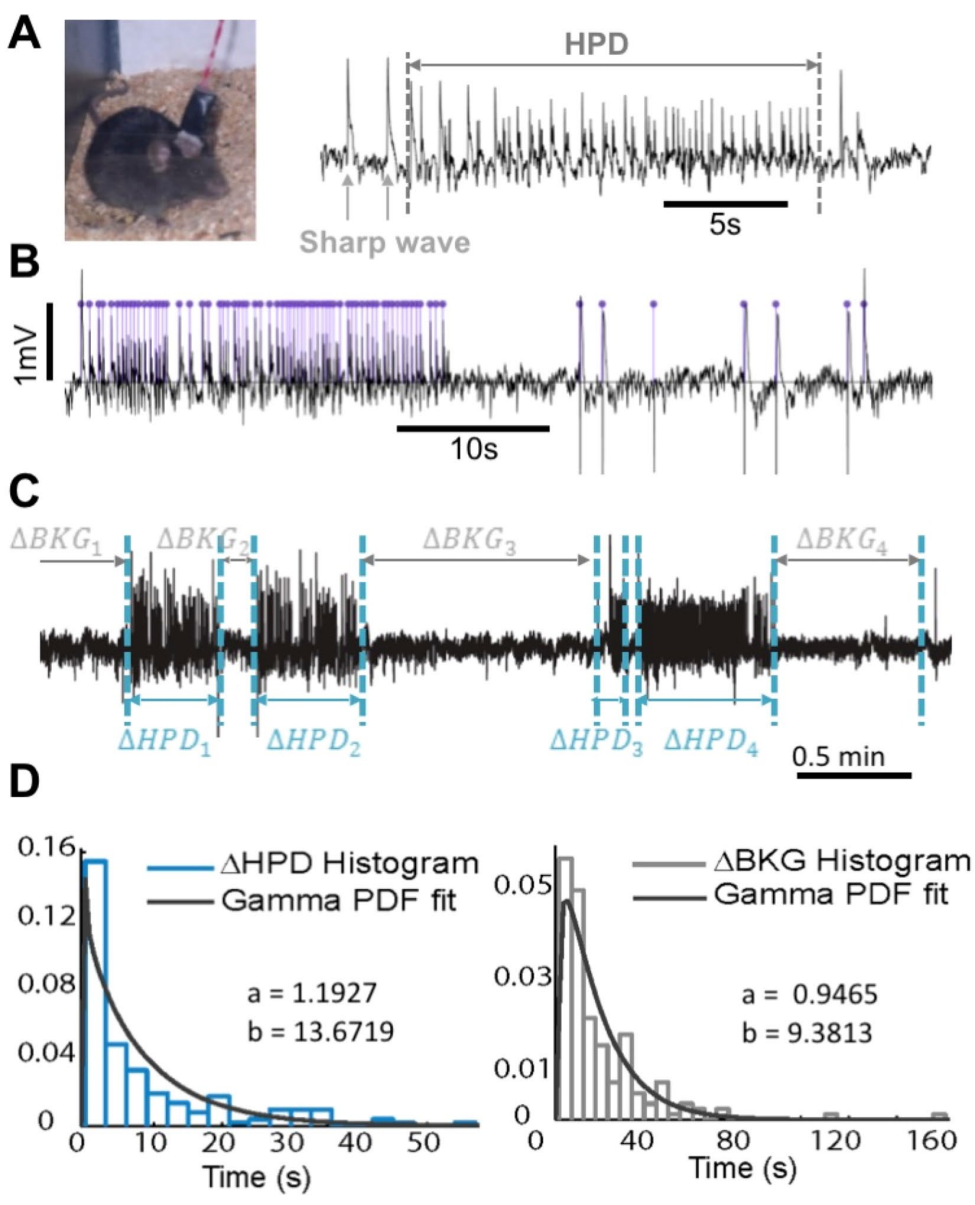
Determination of probability distributions for HPD characteristics. (**A**) *Left*: *in vivo* monitoring of KA mice. *Right*: Typical example of HPD, used in this study as a tissue hyperexcitability marker. (**B**) Example of HPD peaks detection. (**C**) Illustration of the variables used in identifying the probability distributions governing the occurrence rate, inter-HPD duration, and duration of HPD. (**D**) Probability laws fitted from experimental data used to identify the Input Noise Modulation Function are presented in Fig. 5C.

We used the kainate (KA) mouse model^40^ of epilepsy, in which hippocampal paroxysmal discharges (HPD, bursts of high-frequency spikes and sharp waves appearing by the end of the third week post-KA, increasing in duration and occurrence throughout the epileptogenesis phase^41^) are present (Fig. 4A).

### Signal processing

#### HPD detection and characterization

Band-pass filtering (20–80 Hz) was used to increase the contrast between transient sharp events and background activity. The Page-Hinkely test^42^, validated in sporadic spikes detection^43^, was then used (see Fig. 4B) to detect the individual successive spikes in HPD. We developed the following event detection algorithm to detect the onset/offset of epileptic events:

- Each detected peak revealed an ongoing epileptic event starting at least 0.1 s before and lasting 0.4 s after the detected rupture.
- Two consecutive peaks, p1 and p2, were said to belong to the same discharge if their hypothetical time slots (onset-end) overlapped or were separated by less than 0.5 s. This is similar to Heinrich’s definition for HPD classification^41^ (two spikes belong to the same discharge if they are separated by less than 1 s).
- If b. is true, then detected time slots of p1 and p2 were merged into a single slot $[{t}_{onse{t}_{{p}_{1}}}{t}_{en{d}_{{p}_{2}}}]$.

This algorithm enabled the detection of all epileptic event epochs in LFP. Quantifying detected HPD involved computing 1) total duration, 2) total intensity of discharge in a time window (*t*_*win*_) and total number of detected peaks in this time window. Finally, the intensity feature corresponded to the energy of epileptic events in this window:

1 $${E}_{{t}_{win}}=\sum _{i=1}^{D}\frac{1}{N(i)}\sqrt{\sum _{j=1}^{N(i)}{\vartheta }_{j}^{2}}$$

where *D* is the number of detected discharges, *N*(*i*) is the number of samples of the i^th^ discharge, ${\vartheta }_{j}$ is the *j* ^*th*^ HPD.

#### HPD statistical features

Two independent random variables were considered (Fig. 4C) to model the stochastic occurrence rate of HPD of various durations: Δ*HPD* representing HPD *duration*, and Δ*BKG* representing the *inter-HPD duration* as measured between two consecutive HPDs. Measures of Δ*HPD* and Δ*BKG* were calculated from LFP recordings (2 hours) to identify the corresponding statistical distributions. Histograms of the two random variables suggested a possible exponential distribution; however, given that neither HPD duration nor inter-HPD duration can be null, a gamma distribution was chosen, as in a previous study^44^. The probability density function *f*(*x*, *a*, *b*) of a random variable *X* following a gamma distribution is:

2 $$X \sim \Gamma (a,b)\,\,f(x,a,b)=\,\frac{{b}^{a}{x}^{a-1}{e}^{-bx}}{(a-1)!}$$

An exponential distribution was excluded (a = 1). Parameter identification (Fig. 4D) was performed using the Statistics toolbox in Matlab^®^ (GUI fitting tool dfittoll).

#### HPD computational modeling

Two major approaches exist for modeling hippocampal LFP: the ‘detailed’ (microscopic) and ‘lumped’ (mesoscopic) approaches. While the microscopic approach^45–47^ takes into consideration single neurons characteristics, the mesoscopic approach^30, 33^ describes hippocampal dynamics as interacting neural populations. We previously explored both approaches, developing a microscopic detailed^48^ and a mesoscopic lumped parameter model^30^ of the hippocampal CA1 region^49^. The mesoscopic model used here provides access to physiological variables potentially modulated by stimulation (e.g., membrane potential). We used the aforementioned computational model originally developed to describe the CA1 region of the hippocampus to simulate dentate gyrus (DG) activity, since DG basic networks have comparable properties (principal glutamatergic neurons, slow/fast GABAergic interneurons; and synaptic connectivity patterns between neuronal subpopulations).

#### Model Architecture

**Figure 5:**
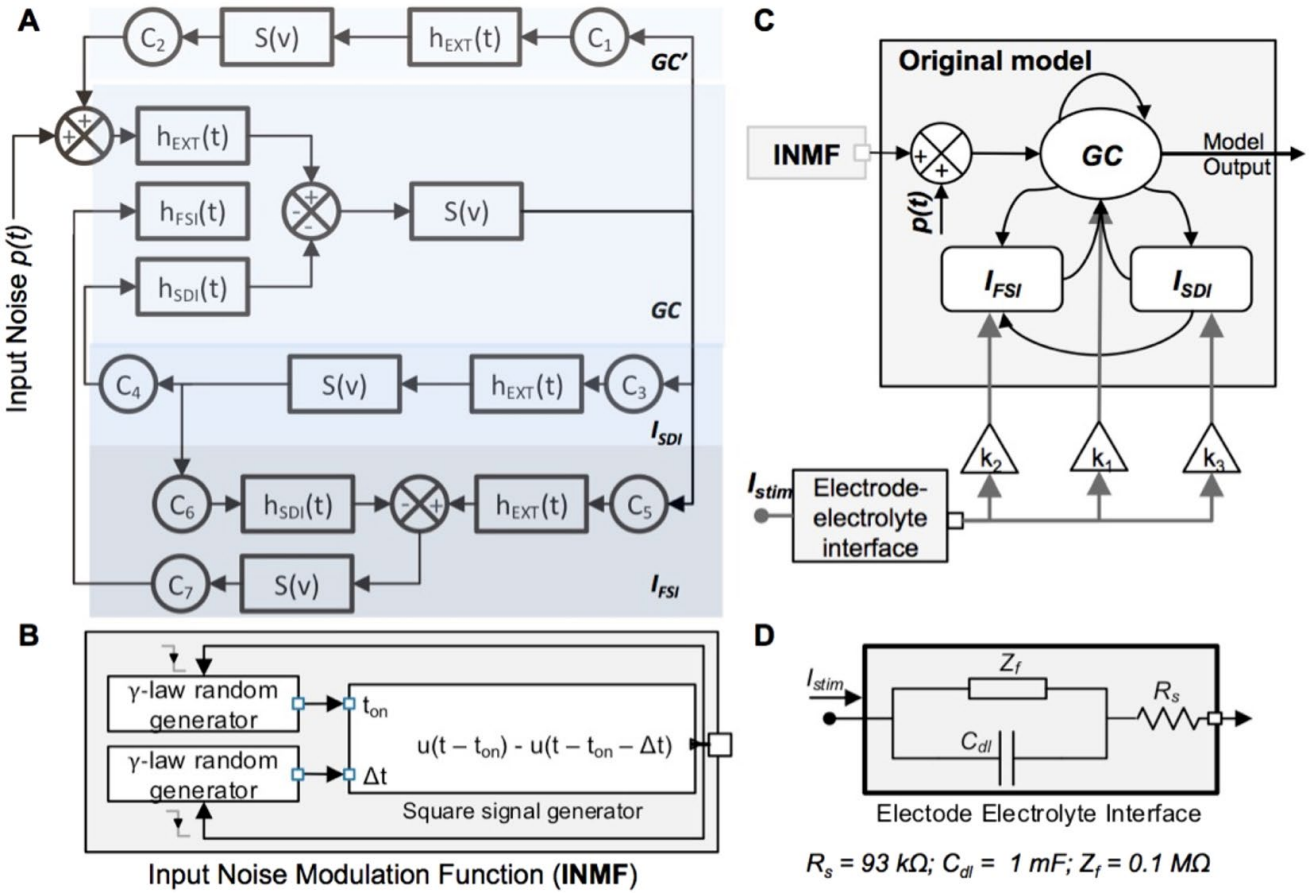
Block diagram of model components. (**A**) Block diagram of the neural mass model, illustrating the considered neural populations and h-functions describing post-synaptic potentials. (**B**) Block diagram of the Input Noise Modulation Function (INMF) for HPD generation. (**C**) Block diagram of the two novel components of the extended neural mass model, including the INMF for HPD simulation, and electrode-electrolyte model with population-dependent coupling coefficients (k_1_, k_2_, k_3_) to simulate LDCS effects. (**D**) Detail of the electrode-electrolyte model.

The model^30^ includes three interacting neural subpopulations (Figs 5A and 2C): a GC cells population; and two interneuron populations, *I*_*FSI*_ and *I*_*SDI*_ (Interneuron Fast Somatic Inhibition and Interneuron Slow Dendritic Inhibition, respectively), representing fast/slow GABA-mediated synaptic transmission, respectively, each represented by input and output transfer functions. The input function (pulse-to-wave function^50^) converts presynaptic action potentials density into an excitatory/inhibitory (EPSP/IPSP) postsynaptic potential, and acts as a linear second order low-pass filter^51^ *:*

3 $$H(s)=\frac{W}{{(s+\frac{1}{{\tau }_{w}})}^{2}}$$

where *s* is the Laplace variable, $W/{\tau }_{w}^{2}$ is the filter’s static gain and $1/{\tau }_{w}$ is the filter’s central frequency. The output function (wave-to-pulse function^50^) converts incoming postsynaptic potentials *v* into a population firing rate *S*(*v*):

4 $$S(\nu )=\,\frac{2{e}_{0}}{(1+{e}^{r({\nu }_{0}-\nu )})}$$

where 2*e*_0_ is the maximum firing rate, *v*_0_ the postsynaptic potential for firing rate of *e*_0_, and *r* the sigmoid steepness. Let us mention that all three subpopulations are necessary for realistic simulation of epileptiform discharges^30, 33^. We developed two model extensions: 1) input noise model modification to simulate HPD generation, and 2) electrical stimulation effects modeling for HPD modulation (Fig. 5).

#### First computational model extension: input noise modulation

HPD are intermittent, sustained interictal discharges that can be described by bifurcations of the neuronal system from fixed point dynamics (background activity) to limit cycle dynamics and back to the fixed point, the time spent at each state being stochastic. We used the aforementioned gamma distributions to modulate DG granule cells input noise in the model, using an Input Noise Modulation Function (INMF, Figs 5B and 2C). Since HPD appear to originate from a subpopulation of cells within the DG (presumably since the KA injection is performed in the DG), we considered a drive from a DG sub-population which triggers HPD. We added stochastic square signals of duration Δ*HPD* (*HPD duration* or *Δt* in Fig. 5B) to the nonspecific input noise *p*(*t*) arriving at GC cells (see Fig. 5C), following this algorithm:

1. Initialization: At t = 0, generate a value for **Δ** ***HPD*** and another for **Δ** ***BKG*** (see section *2*.*1*.*3*).
2. Dynamically simulate the model till t = **Δ** ***BKG***. Between t = **Δ** ***BKG*** and t = **Δ** ***HPD*** + **Δ** ***BKG***, a constant square signal *K* is added to the input noise *p*(*t*).
3. The end of the step *K* triggers the random generator to generate the next values of **Δ** ***HPD*** and **Δ** ***BKG***.
4. Again, between t = t_0_ + **Δ** ***BKG*** and t = t_0_ + **Δ** ***HPD*** ***+*** **Δ** ***BKG***, a fixed step *K* is added to the nonspecific noise *p*(*t*). t_0_ is the instant at which **Δ** ***HPD*** and **Δ** ***BKG*** last values were generated.
5. Back to step 3.

#### Second computational model extension: stimulation effects

Given demonstrated *in vitro* and *in vivo* polarizing effects of electric fields on neuronal elements, stimulation inputs weighted by the stimulation coupling coefficients were summed with mean PSPs before applying the sigmoid transfer function of the stimulated subpopulation. The electrode-electrolyte interface, resulting from metallic electrode insertion in an electrolytic medium, was implemented^38^, accounting for charge accumulation at the interface (see Fig. 5D). A faradaic impedance *Z*_*f*_ in parallel with a double layer capacitance *C*_*dl*_ models the interface between the cerebral tissue and the implanted electrode. *C*_*dl*_ represents capacitive charge injection by stimulation electrodes, while *Z*_*f*_ models the faradaic irreversible charge injection. The solution resistance *R*_*s*_, in series with this interface, represents tissue resistance between the two tips of the bipolar electrode. *Z*_*f*_ and *C*_*dl*_ values depend on electrodes chemical composition (stainless steel in this study), AC-impedance characteristics being well studied as a function of frequency^52–55^. *Z*_*f*_ value was set to 0.1 MΩ and *C*_*dl*_ to 1 mF, resulting in a time constant of 100 s. Finally, considering the grey matter conductivity value of 0.35 S/m, *R*_*s*_was calculated as the resistance between the electrode tips each of diameter 125 µm and separated by 400 µm, resulting in a 93 kΩ value.

#### Electric field distribution and effects on tissue

The effects of the uniform electric field generated by parallel plates are dependent on field orientation with respect to the somato-dendritic axis of neurons^18, 32, 35^, determining whether stimulation hyperpolarizes or depolarizes the membrane. However, the effect of field orientation on interneurons is not as evident, since stimulation effects are related to the type of stimulated interneurons and may be independent of field orientation^32^. Consequently, we considered in our mesoscopic model that the electric field potentially affects differently the three hippocampal subpopulations through stimulation coupling coefficients *k*_*1*_, *k*_*2*_ and *k*_*3*_, modulating stimulation current impact on each subpopulation (GC, fast/slow interneurons, Fig. 5C). The resulting additive membrane potential (Fig. 5C) was different for each neuronal population (dV_1_, dV_2_ and dV_3_, related to k_1_, k_2_ and k_3_, respectively).

**Figure 6:**
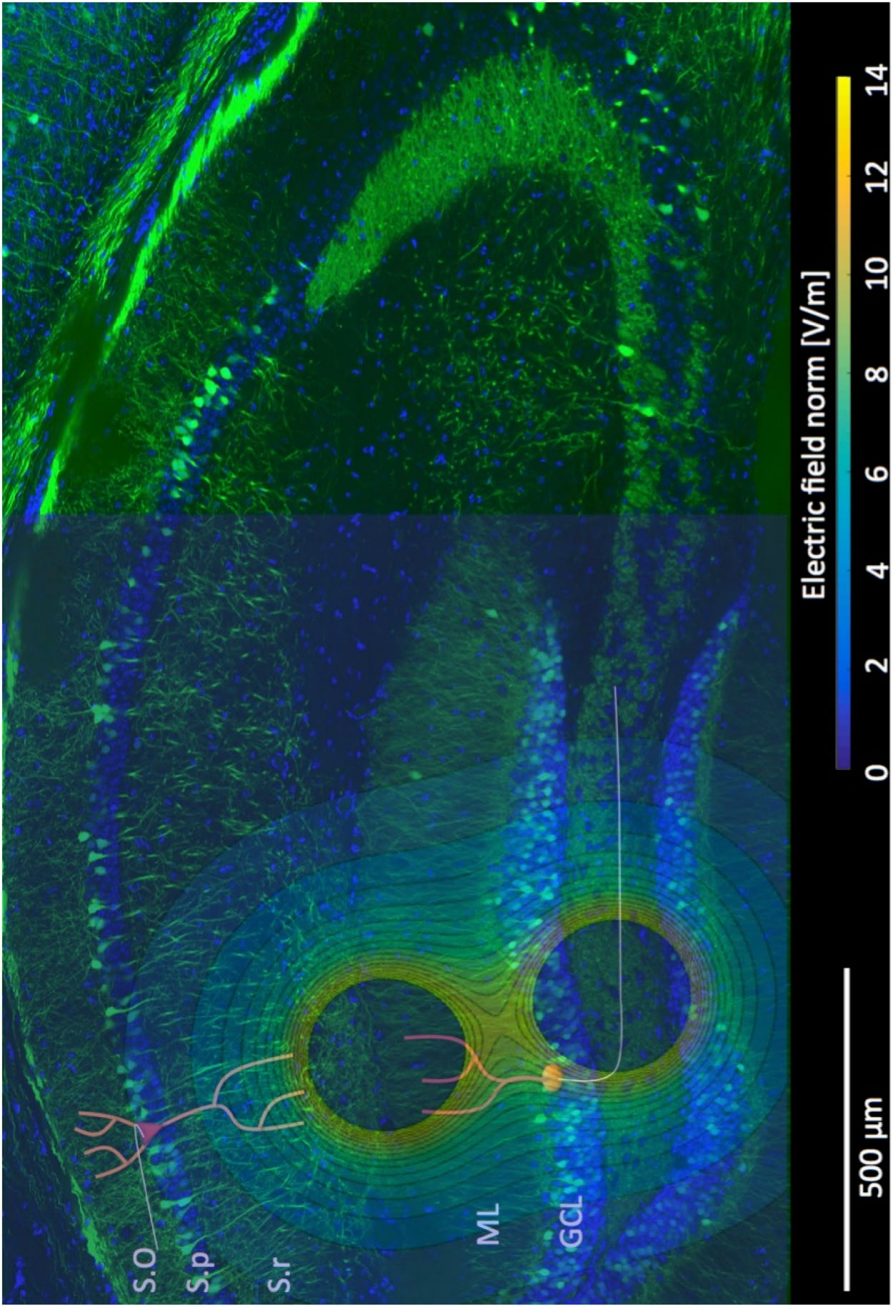
Electric field map. Distribution of the electric field generated by our set of twisted wires electrodes, superimposed on an image of hippocampus, with an example of neuronal orientation for pyramidal cells in S.p and granule cells in GCL. The hippocampal sclerosis due to kainic acid injection is clearly visible. S.O: stratum oriens, S.p: stratum pyramidale, S.r: stratum radiatum, ML: stratum lacunosum-moleculare, GCL: granule cell layer.

We computed the electric potential distribution induced by our specific electrodes (superposition of two potential distributions, each being described by V(r) = I/(4πσr) on a square 2D domain (1 mm^2^). Electrode tips distance was 400 μm, tissue electrical conductivity was σ = 0.35 S/m, and current intensity was 1 μA. We then produced an electric field distribution map with iso-field lines (Fig. 6). The electric field between electrodes reached values (>10 V/m) greater than electric fields induced at the cortical level by transcranial direct current stimulation in humans (0.2–0.3 V/m).

Importantly, as opposed to previous studies^18^ that involved the placement of cathode and anode in distinct brain regions, both cathode and anode were located within the same brain region, i.e. the DG. Our twisted wires electrode featured indeed the cathode and anode 400 µm apart, enabling placing them in the DG for precise targeting of a specific neurophysiological target, i.e. the synaptic terminals of dendrite-targeting GABAergic interneurons. Another key point is that placing the cathode and anode within the same brain region results in a more focal electric field distribution, and therefore stimulation, than placing them in distinct brain regions.

#### Model dynamics analysis

Model dynamics was numerically explored as a function of k_1_, k_2_ and k_3_ to define the boundaries of the analysis intervals [k_1, min_ k_1, max_], [k_2, min_ k_2, max_] and [k_3, min_ k_3, max_]. For each triplet (k_1_, k_2_, k_3_), model output was quantified using HPD characteristics (total duration and intensity per minute), as in our *in vivo* study. In order to quantify stimulation effects on simulated LFP signals, the same characteristics were considered both for simulated and experimentally recorded LFP (detected peaks/minute, discharge intensity and duration). Stimulation effects were analyzed during the minute following the end of the amplifier’s saturation period during which no LFP signals can be recorded.

#### *In vivo* recordings and stimulation protocols

Two experimental validation protocols involved N = 8 epileptic mice to study 1) polarity-dependent effects (n = 3), and 2) time-dependent stimulation effects (n = 5). Experiments were conducted in accordance with the European Communities Council Directive of November 24 1986 (86/609/EEC), and were approved by the ethics committee of Rennes (agreement N° R-2012-PB-Ol). The kainate mouse model (intra-hippocampal kainic acid –KA– injection during a stereotactic surgical procedure) was used^56^ in adult male mice (85 ± 10 days old). The exact coordinates for kainic acid injection within the DG were the following: −2 mm (antero-posterior, AP), −1.5 mm (mesio-lateral, ML), −2 mm (dorso-ventral, DV) from the Bregma, according to the mouse brain atlas^57^. KA injection triggers the epileptogenesis phase (4 weeks). At day 27 post-KA, confirmed epileptic animals underwent a 4-hour baseline recording to quantify basal epileptic activity of each animal before stimulation. Recorded LFPs were sampled at 2048 Hz and hardware-filtered (high-pass, 0.16 Hz cutoff). The same electrodes were used for stimulation and recording.

Upon protocol completion, mice were injected with a lethal dose of chloral hydrate. Brains were removed from the skull and frozen in isopentane (2-methylbutane) at −35 °C. Frozen brains were cut in coronal sections (20 µm thickness) on a cryostat and collected on gelatin-coated strips. Tissue sections were stained using the Nissl staining method to verify (1) electrodes position (2) neural dispersion provoked by KA perfusion and (3) absence of collateral damages or neural death possibly provoked by DC stimulation. The Nissl staining method consists in immersing brain slices in a 0.1% cresyl violet solution for 8 minutes, and dehydrating them in increasing concentrations of ethanol. Finally, slices are cleared through the immersion in two consecutive butan-1-ol baths (8 minutes each), and cover-slipped in a resin layer between two glass strips. Histological evaluation was done using a Nikon optical microscope, enabling verification of electrode position in the hippocampus, as well as KA-induced hippocampal histopathology, and finally possible effects of stimulation currents on hippocampal tissue integrity.

#### Protocol 1: Polarity-dependent stimulation effects

Protocol 1 was tested on 3 adult mice. After a baseline recording for each animal, a stimulation session was performed (GRASS Technologies S88X stimulator) without a priori on electric field polarity. Each stimulation session consisted in two sub-sessions separated by 1 hour without stimulation. Each sub-session consisted in 4 epochs (1 µA during 50 s separated by 300 s for measuring hippocampal response to stimulation, see Fig. 2B). Stimulation current polarity was inverted in the second stimulation sub-session.

#### Protocol 2: Time-dependent effects

Five mice (n = 5) were tested under protocol 2. Tested mice underwent 4-hour baseline recordings around day 27 post-KA before performing a stimulation session. Only cathodal stimulation was delivered in Protocol 2, which consisted in pre-stimulation (1 hour), per-stimulation (1 µA during 50 s every 5 minutes, 11 epochs total), and post-stimulation (1 hour) recordings.

#### Statistical Analysis

HPD duration and intensity were quantified for baseline and stimulation conditions as described in the section “*HPD detection and characterization*”. In order to quantify the imminent polarity-dependent stimulation effects (protocol 1), the value of *t*_*win*_ was set to 1 minute. This time epoch corresponded to the minute following the recovery of the recording electrode after stimulation. The relative discharge duration in each *t*_*win*_ was accounted for as the effect of one stimulation pulse-polarity. For comparability, baseline data was analyzed with the same window length *t*_*win*_.

Given the relatively small population size (<16 per stimulation condition per animal), the Mann-Whitney test is the optimal tool to statistically study the induced effects. It is more stable and robust to outliers when compared to the t-test and is independent of the tested population’s distribution^58^. The statistical significance of polarity-dependent stimulation effects was tested by comparing the change in baseline HPD duration and intensity induced by cathodal and anodal stimulation. The difference between the effects of anodal and cathodal stimulation on HPD duration and intensity were also compared per animal and for the group using the Mann-Whitney test.

Time-dependent effects were also tested for statistical significance using the same approach described above. The analysis window *t*_*win*_ was set to 5 minutes (excluding the 30-second stimulation duration). The effects were compared per animal and for the group.
